# Showcasing the multifaceted aspects of agricultural transformation: The example of mountain oases in Oman

**DOI:** 10.1371/journal.pone.0276580

**Published:** 2022-11-11

**Authors:** Eva Schlecht, Uta Dickhoefer, Shadha Aloufi, Othman Alqaisi, Andreas Buerkert

**Affiliations:** 1 Animal Husbandry in the Tropics and Subtropics, University of Kassel and University of Göttingen, Witzenhausen, Germany; 2 Institute of Animal Nutrition and Physiology, Kiel University, Kiel, Germany; 3 Animal and Veterinary Sciences Department, College of Agricultural and Marine Sciences, Sultan Qaboos University, Muscat, Oman; 4 Organic Plant Production and Agroecosystems Research in the Tropics and Subtropics, University of Kassel, Witzenhausen, Germany; Neijiang Normal University, CHINA

## Abstract

In the Anthropocene the consequences of land-use transformation on ecosystem services are of growing concern, particularly in fragile areas of mountain agriculture that often represent high nature-value farmland. This study uses a decadal repeated survey approach to analyse the effects of modernisation on oasis systems in the Jabal Al Akhdar region of northern Oman. This rugged mountain region at the north-eastern tip of the Arabian Peninsula experiences a growing influx of regional and international tourists since the opening of a modern highway 15 years ago. In 2007, at the onset of transformation processes, a survey was conducted with all households (HH) located in three major settlements along the 1000-m-altitude gradient of the Wadi Muaydin watershed. The survey was repeated in 2018, including all remaining HH of the three settlements. This longitudinal approach allowed studying the consequences of social-ecological transformation processes on crop and livestock husbandry, agricultural labour use, product marketing, and perception of the region’s future by its local residents. Though the village inhabitants are aging and declining in numbers, they still adhere to agriculture, largely because of tradition and identity. Fallowing and abandoning farmland increased over the investigated time span but was paralleled by increased application of agrochemicals and animal manure on fields, purchase of roughage and concentrate feeds for small ruminants, concentration on cash crop and meat production for sale, and increased employment of migrant workers. These indicators of modernisation of oasis agriculture are accompanied by predominantly pessimistic views on future prospects of oasis farming. Commonly perceived problems are shortage of irrigation water and profound societal change. Against these challenges, value chain generation and direct marketing opportunities for local agricultural produce are seen as prerequisites to keep the high nature-value farmland of these mountain oases alive.

## Introduction

In the Hajar Mountains of northern Oman, oases settlements are very particular social-ecological systems [1–3] that have, over centuries, shaped and preserved species as well as habitat diversity [4, 5]. Like other mountainous and insular regions, they can be classified as “high nature-value farmlands” [6, 7]. Such areas serve natural and agricultural biodiversity conservation [5, 8–10] and are defined as anthropogenic landscapes with a considerable share of natural or semi-natural vegetation that host species or provide habitats of high conservation value [11]. While the unique social-ecological value of high nature-value farmlands is recognized in the European Union since more than a decade [12], this is not yet the case in many other regions of the world.

In contrast to a broad range of rather extensive traditional land-use systems of high agro-ecological value reviewed by O’Rourke and colleagues [13], Oman’s irrigated mountain oases and their adjacent natural rangelands can be characterized as moderate-external input, high-intensity farming systems [14, 15]. Typical are a regular application of high amounts of organic matter and nutrients to cultivated land *via* livestock manure [14], reliance on small ruminants’ feed intake at the homestead and on the rangelands [16], intense use of manual labour for maintenance of the dry-stone walled terraces, plot irrigation, cultivation, and crop harvest [17], and judicious application of irrigation water [18, 19]. The highly diverse and thoughtful multi-storey arrangements of annual and perennial crop species that are typical for traditional oases systems [20], sophisticated crop rotation patterns [14], and the conscientious use of fallow periods resulted in exceptionally high water use efficiency [20] and world-record yields of alfalfa (*Medicago sativa* L.) cultivated as a fodder crop [14].

As part of the Hajar Mountains, the Jabal Al Akhdar (“Green Mountain”) region was opened to tourists only in 2006. With its spectacular views on rugged rocky ranges and picturesque villages with ancient dry-stone walled terraces known as “Hanging Gardens”, it attracts a yearly increasing number of regional and international tourists [21, 22]. The visitors enjoy luxury hotels, splendid hiking opportunities, well-equipped picnic grounds on different mountain plateaus, and a pleasant highland climate amidst a hot desert region [23]. Of particular attraction is the Sayq Plateau that towers the region [24]. It overlooks the wide and dissected Wadi Muaydin, an 18-km-long canyon of lime- and claystones that spans an altitude gradient from 1080 to >2000 m above sea level (asl). Along the wadi, the inhabitants of several many centuries-old oasis settlements [21] combine *falaj*-irrigated cultivation of annual and perennial plants [18] with livestock-mediated nutrient and carbon transfers from the oasis-associated rangelands to the dry-stone walled gardens [25, 26]. Rangelands as well as gardens harbor a rich diversity of endemic wild plants [4] and old crop cultivars that provide vast evidence of Oman’s historic trade relations to East Africa and Southeast Asia [8, 27].

Like other high nature-value farmlands in desertic [28] and mountainous regions [13], the agricultural systems on Al Jabal Al Akhdar (henceforth abbreviated “Jabal Akhdar”) are currently experiencing great pressure through water scarcity [23] in combination with climate change effects [29, 30], rural-to-urban migration [1, 28], globalization of inhabitants’ lifestyles, and massive influx of tourists [22] as well as biodiversity loss of field flora [5]. Under similar conditions, such pressures either lead to an intensification and specialisation of agricultural systems or to their abandonment [13]. The latter typically occurs where off-farm income-earning opportunities are available for the young generation [6, 31]. Yet, unlike many rural regions threatened by abandonment [11], Oman’s oasis settlements have benefitted, over the past forty years, from improvement of road transport, telecommunication, healthcare and education infrastructure, and general amendment of living conditions [32]. This reflects Oman’s strategy to slow internal migration by bringing amenities to rural areas, which nevertheless is only partially effective in fostering the maintenance of traditional agricultural practices [2, 6, 11]. Therefore, already a decade ago, Buerkert and Schlecht [33] argued for farmers being paid for the ecosystem services provided by their agricultural systems. They also suggested to create specialty market outlets for local products to generate premium prices, thereby decelerating the abandonment of the traditional irrigated mixed oasis farming systems. A recent analysis of land-use changes in Wadi Muyadin settlements showed that cropland abandonment grew by 3.5% during the period 2007–2018, while cement-walled terraces were built to secure cultivated areas. Although the occurrence of cultivated plant species remained unchanged, many species declined in abundance, whereas a few perennial cash crops with low water requirements increased [23]. These land-use data and crop species inventories point to a modernisation of oasis agriculture on Jabal Akhdar, which merits corroboration by household data in order to comprehend the drivers, trajectories, and future implications of change from farmers’ responses [13] and compare them to transition processes of oases systems and high nature-value farmlands in other regions.

Therefore, an interview-based study accompanied the 2007–2018 land-use change analysis along Wadi Muaydin [23] to record farmers’ accounts of their agricultural activities and learn about their aspirations. Key areas of interest were (i) households’ socio-economic situation, (ii) their crop and livestock management practices including use of agricultural labour, (iii) processing and marketing of agricultural products, and (iv) expectations concerning the future of agriculture at household level.

## Materials and methods

### Study area and agricultural system

**Fig 1:**
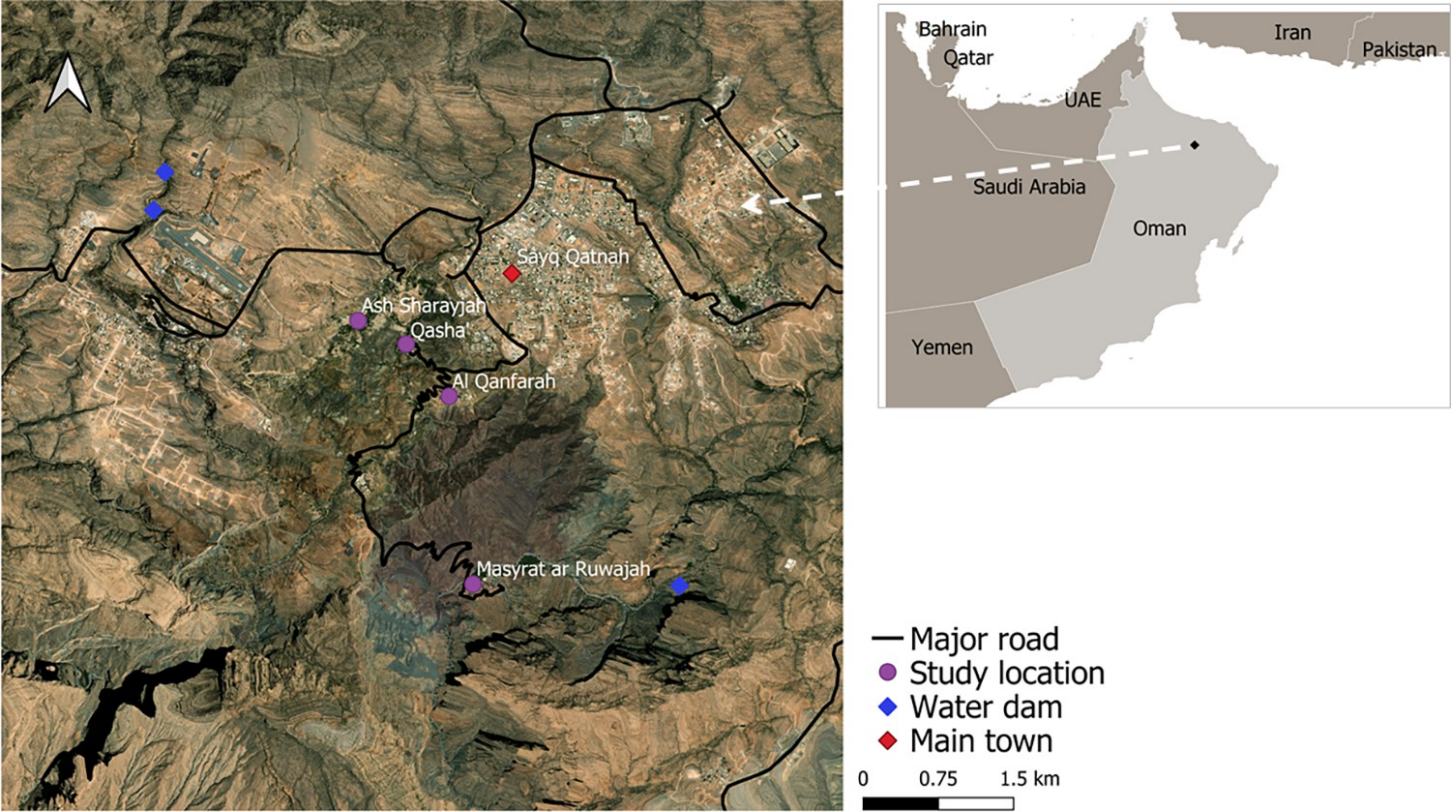
Map of Oman (small insert, upper right corner) and of the study area on Al Jabal Al Akhdar (large map), indicating the studied settlements, the main regional town, and the location of water dams. Source: modified after Google Earth Pro.

The study was conducted in the oasis settlements of Ash Sharayjah (23°04’10” N, 57°39’30” E; 1,900 m asl), Qasha’ (23°04’00” N, 57°39’50” E; 1,640 m asl) with its satellite settlement Al Qanfarah (23°03’43” N, 57°40’03” E; 1,700 m asl), and Masayrat ar Ruwajah (23°02’37” N, 57°40’13” E; 1,030 m asl; Fig 1). Since Qasha’ and Al Qanfarah are situated at the same altitudinal level, we followed the approach of Buerkert and colleagues [23] and treated them together as *Qasha’*, although different water sources are used to irrigate their gardens. While tarmac roads connect Ash Sharayjah (**S**) and Qasha’ (**Q**) with the town Sayh Qatanah (23°22’10” N, 57°36’30” E; 2,000 m asl) on the Sayq Plateau (Fig 1), the gravel road down to Masayrat ar Ruwajah (*Masayrat*, **M**) is long, steep, and winding. In the entire watershed, the irrigated agriculture depends on four major springs feeding two *ayni-aflaj* systems [18, 34]—two springs are surfacing near S, a small one near Q, and the fourth one in the hinterland of M [23]. Due to continuously increasing water demand of sprawling Sayh Qatanah and dwindling spring water availability [23], numerous bore-wells have been installed on the Sayq Plateau since the early 2000s [35], all drawing their water from the same Wadi Muaydin watershed. In 2010, a sewage treatment plant was constructed in S, providing greywater to irrigate the settlement’s terraced land. Treated wastewater from a plant on the Sayq Plateau is furthermore mixed with spring water feeding the interconnected *falaj*-system of Al’Ain, Al’Aqr, and S (M. Al-Rawahi, pers. comm. January 2022). In 2020, a new pipeline for desalinated seawater from Barka at the Omani coast to Sayh Qatanah has started to function, providing drinking water for the population.

In all settlements, agriculture is characterized by the cultivation of human-made, dry-stone walled terraces with irrigated perennial and annual cash crops, food crops, and fodder plants. In 2018, the cultivated terrace area amounted to 16.9 ha in S, 5.6 ha in Q, and 3.4 ha in M [23]. Perennial species vary along the altitudinal gradient, with pomegranate (*Punica granatum* L.) and rose (*Rosa damascena* L.) dominating at high elevation (1,640 to 1,950 m asl), accompanied since recently by olive trees (*Olea europaea* L. [36]). Agriculture in M is characterized by the classical three-storey arrangement of the shading date palm (*Phoenix dactylifera* L.), various medium-sized species of citrus and banana (*Musa* spp.), and understorey fodder crops such as alfalfa, maize (*Zea mays* L.), oats (*Avena sativa* L.), barley (*Hordeum vulgare* L.), and Rhodes grass (*Chloris gayana* Kunth). In the higher-altitude oases, these fodder crops are also cultivated under the canopy of pomegranate and *Prunus* spp. Unshaded plots of all settlements carry cash crops like garlic (*Allium sativum* L.) and vegetables [30].

Until today most households (HH) combine crop cultivation with livestock husbandry; goats of the local Jabal Akhdar breed dominate in numbers, but sheep, cattle, chickens, and rabbits are also kept by some families [37]. During daytime, goats and sheep graze the valleys, mountain slopes, and plateaus surrounding the settlements, while cattle and rabbits are stall-fed with purchased, cultivated, and collected fodder. Chickens are either scavenging in the village or kept within the courtyards [38]. Next to meat, milk, and eggs, the animals’ manure is also an important product for the farm as its application ensures the fertility of the terraced land [1].

### Data acquisition

In spring 2007 and in spring 2018, a participatory survey was conducted at the three locations. It addressed HH characteristics (demography, education, income sources, physical assets), cropping system (area managed, crops cultivated, inputs used), livestock keeping (species and numbers, feeding practices), use of agricultural labour, crop and livestock sales (including product processing and marketing), and major problems as well as opportunities of crop and livestock farming. Prior to conducting the survey, the underlying questionnaire was discussed with senior colleagues at Sultan Qaboos University, who alleged that it met the ethical and cultural requirements for interviews in Oman. Questions were fully compliant with national and local norms, values and traditions and did not allow identifying human subjects or disclosing private information. After its general approval, the survey’s purpose was explained to all interviewees; they were assured that participation in the survey was voluntary, anonymous, not related to any government activities, and that participation or avoidance would not reap any benefit or disadvantage. An interview lasted on average 40 minutes; it only started if oral consent was given by the HH head who was male in all but one case in 2007 and 2018. Only thereafter, the individual interview was conducted with the HH head in Arabic language; the entire conversation took place in privacy within the family home or courtyard, to avoid any real or perceived pressure of third parties. In 2007, interviews were conducted by one female and one male, in 2018 by two female enquirers. Each time one person was of Arabic and one of European background. Answers were translated into English, either directly (2007) or upon entry into the database (2018). While in 2018, questions were modified after a pre-test with 5–7 HH residing in the settlements of Al’Ain and Al’Aqr that were not covered by the survey, phrasing but not content of questions was slightly altered as interviews progressed in 2007.

### Data analysis

The interview data was entered in an Excel® database, coded, and inspected for completeness of records, plausibility of answers, as well as normal distribution of the residuals of continuous variables by using the Kolmogorov–Smirnov test. Statistical analysis was computed using SAS/STAT 15.2 (SAS Institute Inc. Cary NC, USA 2020). Since total garden area, diversity of cultivated perennial species, reliability of water supply, closeness to the town of Sayh Qatanah with its tourism sector, and road infrastructure differed with altitude, being mostly more advantageous in S compared to M and intermediate in Q (see above information), it was assumed that modernisation was more likely to occur in S than in M and Q. Since these factors were nested, they were represented by the variable “village”. For normally distributed continuous variables, the effects of the fixed variables year and village were tested separately by using the *proc mixed* procedure. Differences between villages were determined by the Tukey post-hoc test. For not normally distributed continuous variables, the Mann-Whitney-U test (effect of year) or the Kruskal-Wallis test (effect of village) were applied; the (Fisher’s exact) Chi-square test was used for categorical variables, followed by Wilcoxon signed rank-statistics to discern differences between villages. Significance was declared at P ≤ 0.05 with P-values < 0.1 interpreted as tendencies. Village-specific results are only reported in case of significant differences for a dependent variable.

Unless mentioned otherwise, results for binomial and categorical variables are reported in absolute (n) or relative (%) frequencies for 2007|2018, whereas results for continuous variables are depicted as arithmetic means ± one standard deviation. When respondents were asked to rank a variable such as their first, second, and third most important crop, the absolute frequency of mentions of a specific crop at the particular rank was multiplied with 3, 2, and 1. By dividing the sum of products by the number of valid responses, the weighted importance of the specific crop was obtained, which was then translated back into an ordinal rank number.

## Results

### Household characteristics and income sources

While in 2007, a total of 40 HH participated in the survey, in 2018 only 29 thereof remained for a visit; some interviewees were still residing in their original settlement, others had moved to the nearby towns of Sayh Qatanah or Birkat al Mawz. Responses of two and one HH in 2007 and 2018 were incomplete; therefore the final data set comprised 38 HH and 28 HH for 2007|2018, which were distributed across the villages as follows: S 17|10, Q 10|10, and M 11|8. In 2007|2018, 18%|4% of the HH comprised one generation, 47%|17% comprised two, 34%|72% comprised three and 0%|7% comprised four generations, with a similar average number of generations per HH in the two years and three villages (P > 0.05 in both cases). Average HH size was 10±6.9 in 2007 and 11±5.3 in 2018 (P > 0.05), with the largest HH encountered in Q (P ≤ 0.01), both in 2007 (15±7.3) and in 2018 (14±6.6). In 2007, the average age of HH heads was 54±16.1 years, whereas it was 59±16.1 in 2018 (P > 0.05). Across both years, 89% of HH heads were married, 4% were single, the rest widowed.

All HH were engaged in agriculture, mainly because of family tradition (47%|73%), contribution of agriculture to the family’s livelihood (31%|23%), and income generation (22%|3%), with no differences between years (P > 0.05). Main income sources were government jobs and pensions (87%|96%), while agriculture (11%|4%), an own business (3%|25%), and a son’s salary (13%|0%) were of little relevance. Off-farm income was earned by 1.9±1.64 HH members in 2007 and increased to 3.4±2.47 members in 2018 (P ≤ 0.01), with highest numbers of off-farm income-earning persons per HH in Q (P ≤ 0.01), namely 3.1±1.97 in 2007 and 4.6±3.06 in 2018. Across villages, number of HH members earning off-farm income reduced to 1.4±1.69 in 2007 and 0.68±0.55 in 2018 (P > 0.05) when excluding pension-earners.

As far as assets were concerned, a car was owned by 58%|57% of HH in 2007|2018, a washing machine by 74%|96%, a TV by 87%|93%, a phone by 79%|89%, and a bank account by 82%|96%. Expenditures for food and clothes ranked first in 2007, followed by expenses for agricultural activities including hiring labour, and in third position, maintenance or improvement of the family’s house. In 2018, maintenance or improvement of the house ranked first, followed by expenditures on agriculture, while expenditures for food and clothes, a car, or school fees were of minor importance.

### Challenges and opportunities of crop cultivation

The average size of farm land owned by a HH did not change during the 11-year study period but varied slightly between the three villages (P > 0.05), whereby not all farmers disclosed the area owned. Reported farm sizes were highest in S (10,292±7,802 m^2^, n = 15), intermediate in Q (4,968±6,690 m^2^, n = 10), and lowest in M (2,797±2,090 m^2^, n = 12). Most farmland was located close to the house, at a walking distance of 14±10.4 minutes across villages. In 2007|2018, 15|13 farmers had leased small areas of land from another farmer, and 3 farmers rented out some land to others in each of the two study years.

The share of HH that reported an increase in their cultivation of pomegranate and temperate (S, Q) or tropical (M) fruit trees was close to 40% in both survey years. Concerning their tangible or intangible importance to the household, farmers in S and Q ranked the cultivation of pomegranate (for sale of individual fruits) and rose (for sale of flowers or rosewater) first and second among all crops, whereas cultivation of date palm (for family consumption or sale of dates) was most important for farmers in M in both years. In 2007, a wide variety of fruits other than pomegranate were cultivated in S and Q, such as peach (*Prunus persica* (L.) Batsch), apricot (*Prunus armeniaca* L.), and walnut (*Juglans regia* L.); in M, citrus trees, papaya (*Carica papaya* L.), and banana complemented the dates. The cultivation of these additional fruits was ranked third (S, Q) and second (M), respectively, with fodder crops being ranked third in M. In 2018, the important crops had not changed in S and Q, but vegetables ranked second after dates in M, followed by other fruits. Fodder was no longer listed among the three most important crops in 2018.

In 2007|2018, 34%|54% of the farmers left some of their land fallow; machinery for plowing, tillage, or spraying was used by 50% and 61% of HH in 2007 and 2018 (P > 0.05 in all cases). Trees were pruned by 82%|96% (P = 0.06) and pest control practiced by 68%|79% (P > 0.05) of HH in 2007|2018. While mineral fertilizer was only used by 29%|50% of farmers (P > 0.05), 95%|100% of them used animal manure in 2007|2018, whereby 61%|68% purchased manure in addition to what was collected from their own animals (P > 0.05 in both cases).

Thirteen out of 38 farmers in 2007, but none in 2018, confirmed that they did not face any problems in their cropping activities (P ≤ 0.001). Of the 25|28 farmers encountering problems, water shortage (35%|52%; P ≤ 0.001), high workload (32%|2%; P ≤ 0.01), and prevalence of crop diseases (22%|45%; P ≤ 0.001) were mentioned most frequently in at least one of the two survey years. When asked which crops (up to three) they would cultivate in case they could access more land and water, the responses of 23 farmers pointed to vegetables (31% of mentions), fodder (20%), pomegranate (18%), rose, and date palm (each <10%) in 2007. In 2018, all 28 farmers favored fruits other than pomegranate or date (50% of mentions), followed by vegetables (19%), pomegranate (16%), and rose (12%), with <10% of mentions each of date, olive, and fodder.

### Purposes and practices of livestock keeping

**Fig 2:**
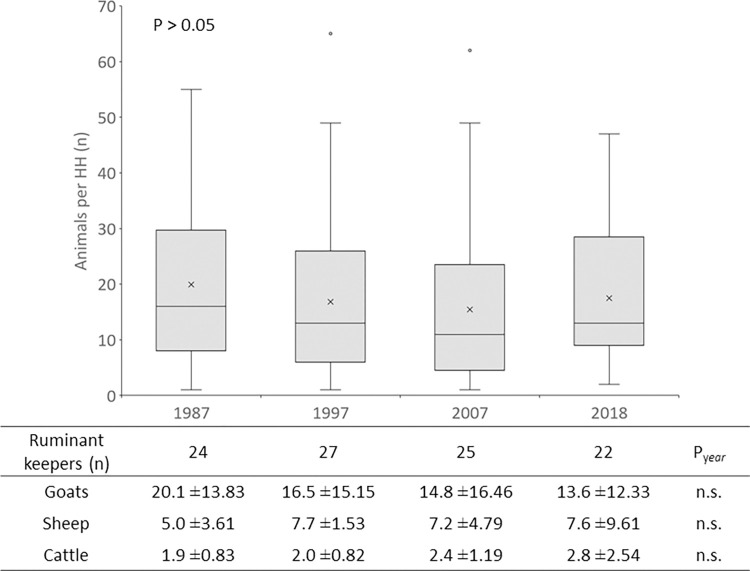
Decadal variation in the total number of ruminants (boxplot, above) and number of goats, sheep and cattle (table, below) owned by ruminant-keeping farm households in the villages Ash Sharayjah, Qasha’ plus Al Qanfarah and Masayrat ar Ruwajah on Al Jabal al Akhdar, northern Oman. In the boxplot, the lower and upper limits of boxes depict lower and upper quartiles, the horizontal line indicates the median, the tick the mean, and the lower and upper whiskers the minimum and maximum, respectively. Outliers are marked by dots.

Animals were kept by 27|26 HH in 2007|2018, with 89%|88% thereof keeping goats (P > 0.05), 22%|46% sheep (P = 0.051), and 48%|65% cattle (P ≤ 0.01). Chickens were kept by 19%|65% of the livestock-keepers in 2007|2018 (P ≤ 0.001), with 0.9±2.20 and 8.6±9.03 birds per HH (P > 0.05). Goats dominated the livestock population (76.5%|61.8%), followed by sheep (13.1%|21.6%) and cattle (9.4%|16.7%), whereby the relative decrease in the importance of goats over time was also reflected in recall data on the variation in ruminant numbers since 1987 that was collected in the 2007 survey (Fig 2).

**Fig 3:**
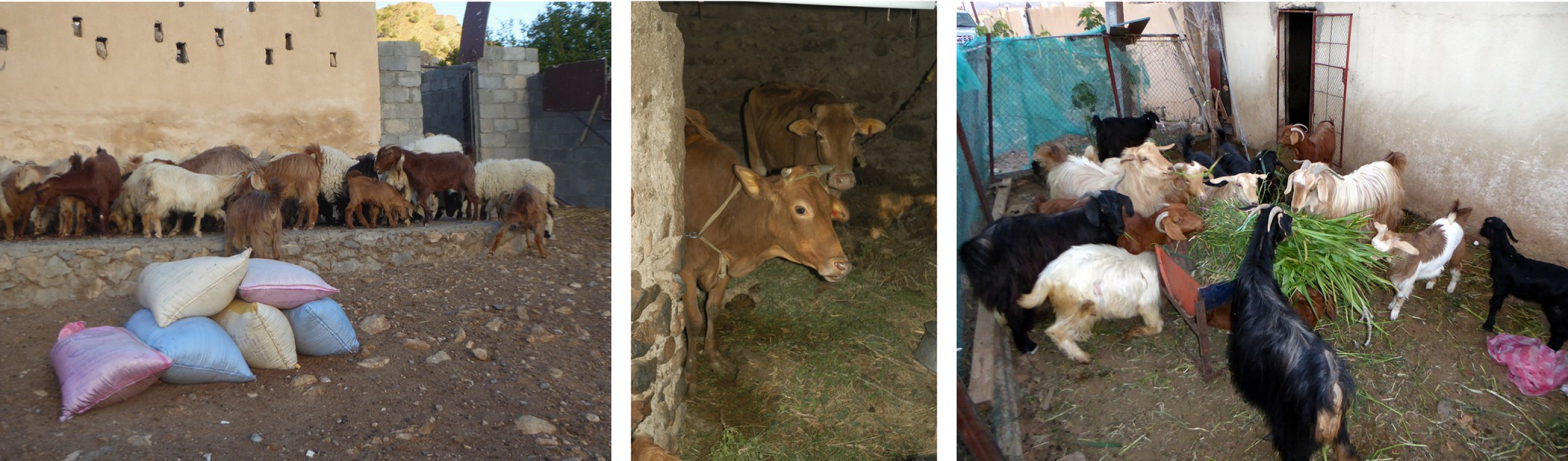
A goat herd and bags with collected goat manure for application in oasis gardens of Al Jabal Al Akhdar, northern Oman, in 2018 (left); cattle kept in the basement of a traditional oasis house in 2007 (center); goats fed cultivated Rhodes grass at the homestead enclosure in 2018 (right).

While the main purpose of keeping animals was meat consumption and income generation, goat milk and hair, respectively, were used by 96%|22% (P ≤ 0.001) and 21%|0% (P ≤ 0.05) of the goat-keeping HHs in 2007|2018. The number of goats sold per HH and year averaged 6±7.3 (n = 9) in 2007 and 9±8.9 (n = 9) in 2018 (P > 0.05). The annual number of goats purchased per HH, to either increase flock size, replace culled animals, or for direct consumption was 5±2.6 (n = 7) in 2007 and 4±3.1 (n = 5) in 2018 (P > 0.05). Small ruminant manure was applied on own farmland by 23|22 HH; only one HH also sold small ruminant manure to neighbors in 2007. The number of collected and applied bags of approximately 25 kg dry small ruminant manure (Fig 3, left) averaged 59±64.0 (n = 23) per HH and year in 2007 and 118±118.2 (n = 22) in 2018 (P ≤ 0.05).

While in 2007, only 8 HH kept cattle, 17 were doing so in 2018. Of these, 31%|12% milked lactating cows (P > 0.05), and sale of on average one head of cattle per year was confirmed by 100%|6% of these HH (P ≤ 0.001). Purchase of 1.7±0.5 heads of cattle per year in 2007 and of 2.2±0.7 cattle in 2018 (P ≤ 0.05) was acknowledged by 67%|73% of the livestock keepers. Cattle not used for milk production were either stall-fed for some months (Fig 3, center) or directly slaughtered and consumed during festivities. Faeces of cattle were dried and collected into bags for application on farmland; this was confirmed by 38%|88% (P ≤ 0.01) of cattle keepers who gathered 27±60.8 (n = 5) and 54±101.5 (n = 15) bags per HH and year (P > 0.05) in 2007|2018.

**Fig 4:**
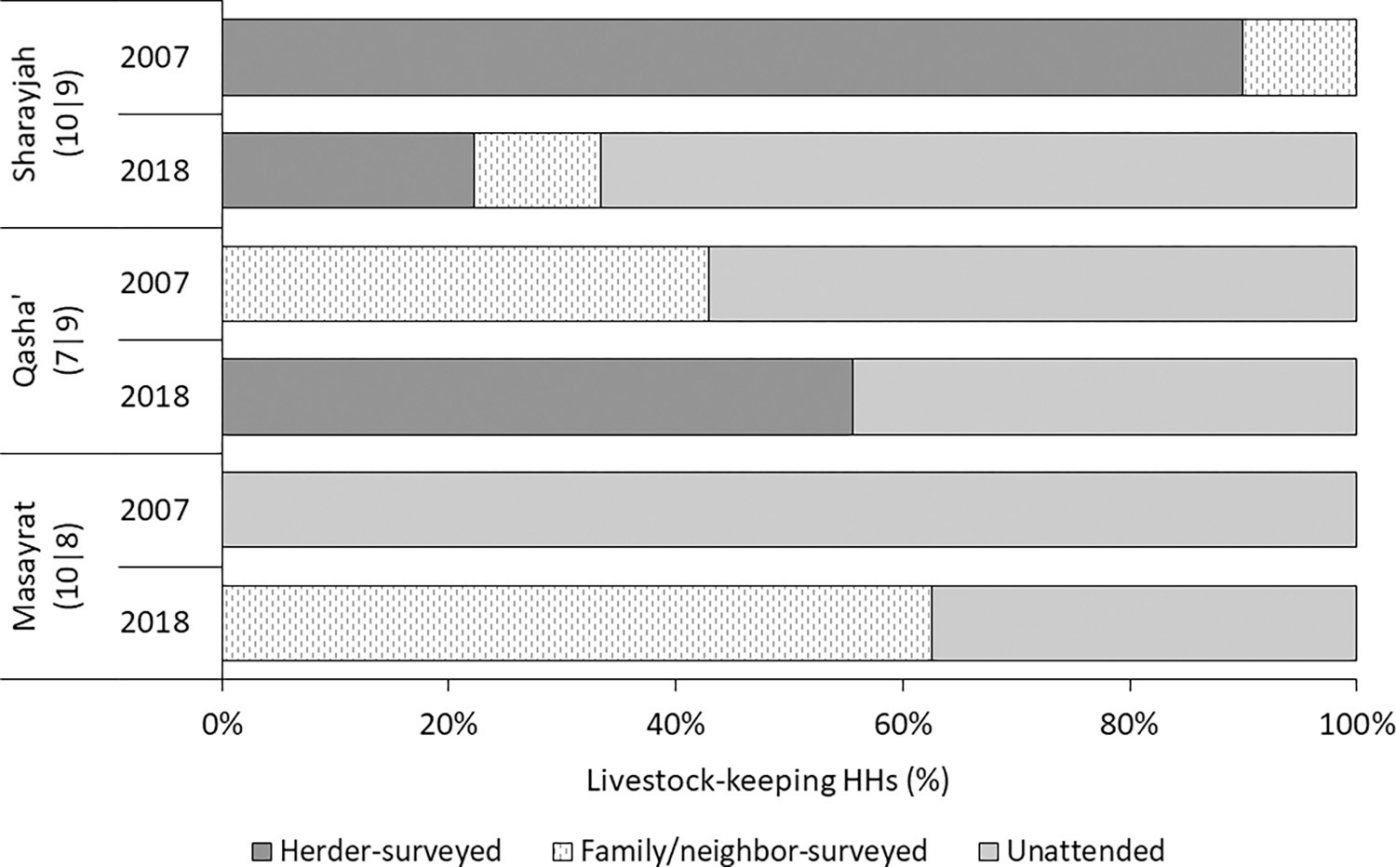
Responsibility for and mode of attending to small ruminants during daily time on pasture in the villages Ash Sharayjah, Qasha’ plus Al Qanfarah (Qasha’) and Masayrat ar Ruwajah on Al Jabal Al Akhdar, northern Oman, in 2007 and 2018. Differences in herding mode were insignificant between years (P > 0.05), but significant between villages (P = 0.018), with significant (P ≤ 0.001) year x village interactions.

While 56%|38% (P > 0.05) of the livestock keepers had constructed a separate animal house or stable for their ruminants (Fig 3, right), the remaining HH kept them in the basement of their houses during night. To feed their ruminants, farmers (85%|100%; P ≤ 0.05) cultivated various fodder plants, purchased fresh or dry Rhodes grass (22%|35%; P > 0.05), and alfalfa hay (15%|0%; P > 0.05) as well as energy-rich concentrate feed and/or old bread from local bakeries (44%|69%; P > 0.05) and dates (96%|62%; P ≤ 0.01) plus protein-rich sardine fish (93%|58%; P ≤ 0.01). In addition to homestead feeding, small ruminants were sent to pasture by 89%|85% (P > 0.05) of the livestock keepers. Thereby, they were either left on their own as soon as they reached the village borders (unattended) or were, for several hours per day or the full day, surveyed by a hired herder or a family member and/or neighbor (herded), with variations in herding practice between villages and over time (Fig 4).

Overall, 19 of 27 (2007) and 5 of 26 (2018) livestock keepers perceived no specific problem in their herds, while the remaining ones highlighted animal diseases (31%), feed shortage, input costs, and herd surveillance (each 15%) as critical issues in 2007. Less important problems were water shortage (for fodder cultivation), the dirty work of cleaning animal houses, and the high workload of livestock care (each 8%). In 2018, only diseases (62% of mentions) and feed shortage (27%) were relevant, while water shortage, lack of vaccines, and livestock predation by feral dogs were rarely mentioned (4% each). Asked about future plans concerning small ruminant keeping, 63%|81% of the livestock keepers confirmed that they would like to expand the herd whereas 37%|12% aimed to maintain its size, and 0%|8% intended to reduce their animal numbers.

### Agricultural tasks and labour allocation

When asked to rank the three most labour-demanding tasks in crop and livestock farming, tree maintenance (manuring, pruning, pesticide application, and hand-pollination of dates) ranked first in 2007, followed by irrigation tasks (cleaning and repair of canals, actual water application), and third by cropping tasks (fertilization, seedbed preparation, sowing or planting, weeding). Concerning livestock, the most labourious task was fodder harvest and actual feeding, followed by herding of animals on pasture, and third by cleaning the animal houses, which included manure collection into bags for transport to the farmland. In 2018, among the labourious cropping tasks tree maintenance shifted to rank four, preceded by crop cultivation (rank 1), irrigation (rank 2), and harvesting of annual and perennial crops (rank 3). Among the labourious livestock tasks, supervising the animals’ grazing advanced to rank 1, followed by the monitoring of hired herders, and, in third position, collection of feed and feeding.

With respect to employing labourers, of the 33 HH responding to this question in 2007, 14 never hired workers and 19 occasionally employed labourers for agricultural work. In 2018, only 3 HH did not hire labourers, 19 HH employed one, 5 HH employed two, and 1 farmer employed seven persons, on a monthly to semi-annual basis. Benefitting from free housing, the monthly salary of these male labourers from Bangladesh, Pakistan, and India ranged from 52 to 100 Omani Rial (1 OMR = 2.60 US $ in March 2018; https://www.exchange-rates.org/Rate/OMR/USD/3-1-2018), with a mean of 79±15.5 OMR (n = 23, median 70) in 2018.

### Product processing and marketing

**Fig 5:**
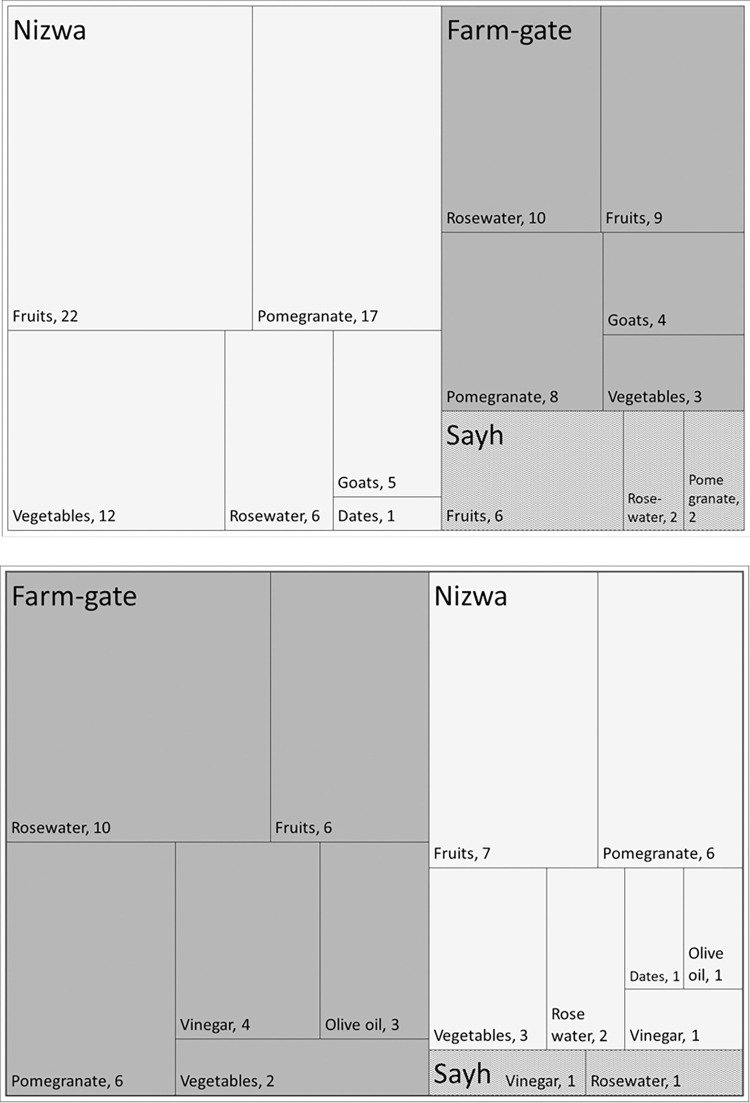
Absolute and relative importance of the marketing outlets farm-gate, Nizwa (farmer market) and Sayh Qatanah (Sayh, shops) used by farm households in Ash Sharayjah, Qasha’ plus Al Qanfarah and Masayrat ar Ruwajah on Al Jabal Al Akhdar, northern Oman, in the years 2007 (top) and 2018 (bottom). The size of an individual branch in the tree map depicts the relative importance of an outlet for a specific product; the numbers identify the total number of farmers selling the respective product via the respective channel.

For 13|13 respondents, processing of plant products was important in 2007|2018 and targeted roses that were processed into rosewater by 13|12 HH, as well as grapes, of which vinegar was produced by 0|5 HHs. The introduction of olive trees in 2014 was supported by governmental advice but completely relied on private investments. This went along with the possibility of processing olive oil at a government mill in Sayh Qatanah (M. Al-Rawahi, pers. comm. on May 2021), but only 3 of 28 respondents used this possibility in 2018. Out of the 38 and 28 HH, respectively, 66% and 54% sold crops, live animals, or processed products in 2007|2018 (P > 0.05). Among the active sellers, 52%|67% had abandoned the sale of one or up to five types of products recently. Commodity sales targeted neighbors, local residents, day-tourists, hotels, and retailers, and primarily occurred at farm-gate (72%|80%; P > 0.05). Sales to food shops in Sayh Qatanah (24%|7%; P > 0.05) were less important than sales at the weekly farmers’ market in Nizwa (96%|53%; P ≤ 0.01), which is the regional center of the Ad Dakhiliyah governorate to which the Jabal Akhdar region belongs. One to four (median 2) types of commodities were sold per farmer *via* any one of the channels in 2007 and 2018, whereby only farm-gate sales increased to a median of 2.5 different commodities per farmer in 2018 (Fig 5). Of the HH selling produce, 52%, 20%, and 8% in 2007, and 47%, 20%, and 27% in 2018 prioritized the farm-gate option, Nizwa, and Sayh Qatanah, respectively (P > 0.05 in all cases). Reasons for the preferences were lower effort for farm-gate sales and better commodity prices at Nizwa, while selling to shops in Sayh Qatanah was on the one hand yielding slightly better prices than farm-gate sales but on the other hand requiring more labour and time (packing, transport). Problems of product marketing were mentioned by 20%|27% (P > 0.05) of the selling HH, with transportation to market and low profit being perceived as main hindrances.

### Farmers’ perceptions of the future of oasis agriculture

**Fig 6:**
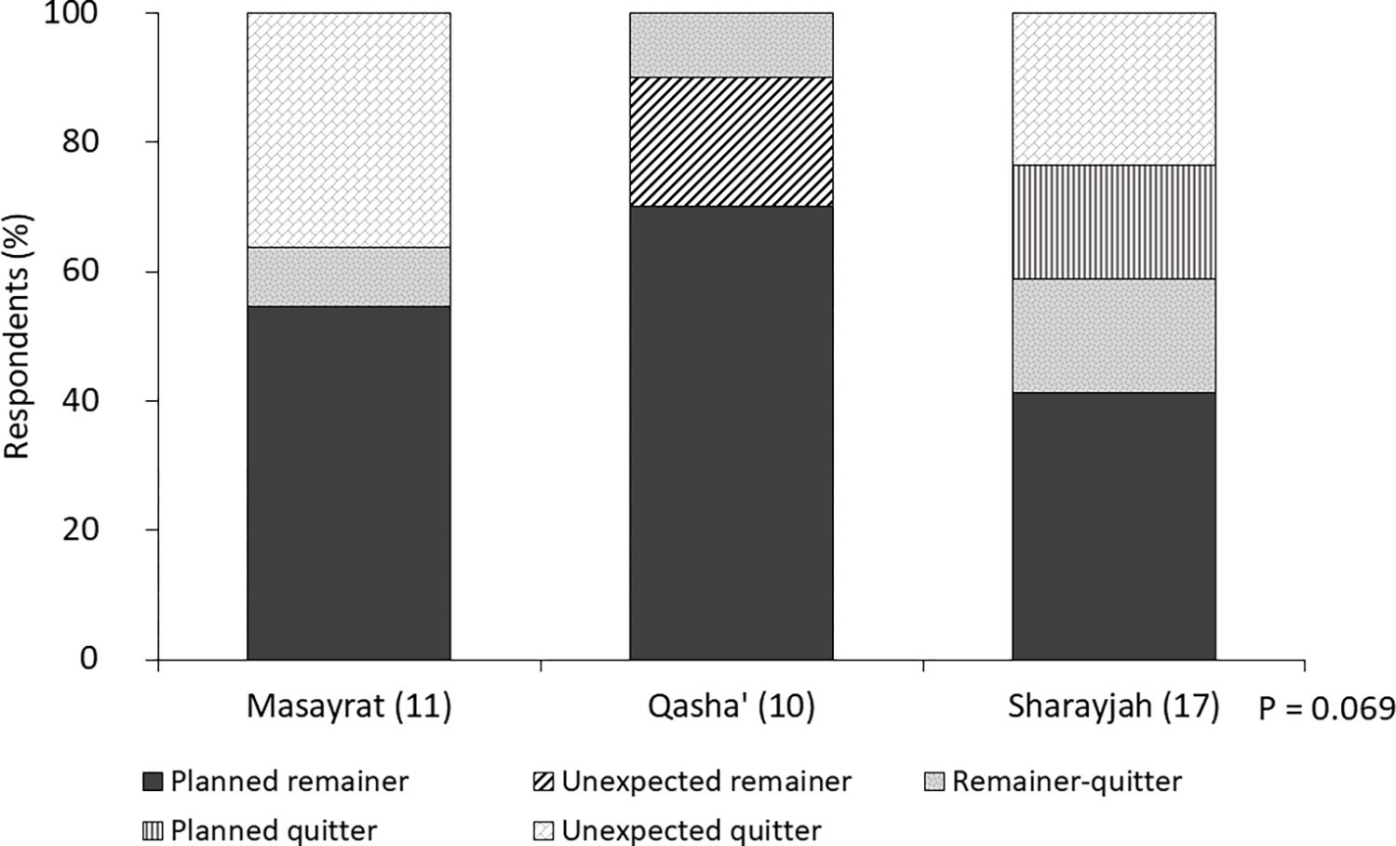
Evolution of farming livelihoods of 38 respondent households in Masayrat ar Ruwajah (Masayrat), Qasha’ plus Al Qanfarah (Qasha’) and Ash Sharayjah (Sharayjah), on Al Jabal Al Akhdar, northern Oman, from 2007 until 2018. A *planned remainer* had planned to continue farming in 2007 and was still actively involved in agriculture in 2018; an *unexpected remainer* had expected to abandon farming in 2007 but was still actively involved in 2018; a *remainer-quitter* had planned to continue agriculture in 2007 and was still involved in 2018 but expected to abandon the activity soon; a *planned quitter* had planned to abandon farming in 2007 and had left agriculture in 2018; an *unexpected quitter* had planned to continue farming in 2007 but had left agriculture in 2018.

When asked if they would in any case continue farming as a livelihood activity, 87%|82% of the 38|28 HH confirmed, whereas 13%|18% denied; these numbers hardly changed when respondents, in both years, were asked if they continued farming in case agricultural income opportunities would improve. Asked if they anticipated that their children would continue farming, 68% of the respondents were affirmative in both years, 16%|25% were dissenting, and 19%|7% were unsure. The dropout of 10 of the initially interviewed HH (26% of 38) during the 11-year study period proved that the respondents’ assessment of their family’s future as a farming HH had been realistic in 2007 (Fig 6). Thereby, the share of HH abandoning agriculture was highest in S, followed by Q and M (P = 0.069). At 38%, the share of dropouts was higher (P > 0.05) for HH that had ≤ 10 members in 2007 (n = 21) compared to 18% dropouts for HH that had > 10 members (n = 17) in 2007. Similarly, among HH managing ≤ 3000 m^2^ of farmland in 2007 (n = 16), a higher share (38%) abandoned agriculture (P > 0.05) compared to HH with medium-sized farmland in 2007 (3001–9000 m^2^; 29%; n = 14) and with large-sized farmland in 2007 (> 9000 m^2^; 13%; n = 8).

In 2007, only 24 but in 2018 all 28 interviewees agreed to discuss the future of the Jabal Akhdar region. Of these, 67%|46% (P > 0.05) were quite or very optimistic about the regions’ economic situation, 13%|50% (P ≤ 0.001) did rather not expect an improvement, and 21%|4% (P ≤ 0.001) were unsure. Naming up to three problems per respondent, poor transportation, housing, and communication infrastructure were mentioned frequently in both years (44%|36%), along with shortage of irrigation water (12%|39%). In addition, outmigration of local residents, increasing insecurity, and unemployment were of concern to some respondents in 2007, whereas a negative impact of tourism, the influx of migrant workers, and lack of crop land were occasionally mentioned as critical issues in 2018. Higher incomes, more off-farm income-earning opportunities, as well as better infrastructure were considered important for the regional development in both study years.

## Discussion

### Socioeconomic development in the oases settlements

For many centuries, Oman’s mountain oases have provided their inhabitants with major ecosystem services and were locally important economic and cultural centers [17, 39]. In 1978, S, Q, and M had been inhabited by about 290, 150, and 230 persons [40]. These numbers changed to 214 (S), 145 (Q), and 111 (M) in 2001/02 [41] and to 140 (S), 152 (Q), and 127 (M) in spring 2007 [37]. Until 2008, three more families from S, two from Q, and three from M left [37], and until 2018, another seven families from S moved to Sayh Qatanah, three families from M moved to Birkat al Mawz, whereas no further changes occurred in Q. The 2018 interviewees provided evidence that all of the ten HH that had left the settlements since 2007 still cultivated their farmland during occasional week-end visits or through hired farm workers. The outmigration of entire families can in part be explained by limited construction space in the mountain villages and high transport costs for building materials, especially to the more remote locations of Q and M. This is underlined by the highest importance rank given to expenditures for house construction and maintenance in 2018. Especially for families from S, the move to Sayh Qatanah seemed to be a straightforward decision, which is mirrored in the high share of HH in S that abandoned their agriculture-based livelihood during the period from 2007 to 2018. As a consequence of high intraregional mobility, the town of Sayh Qatanah has expanded massively during the last decade [23].

The average HH size of 10 members in 2007|2018 was only half of the 21 heads reported for 1978 [40]. While only 33% of HH in the three locations comprised more than two generations in 2007, this share increased to 43% in 2018, which, together with the increasing average age of HH heads, points to a substantially skewed age distribution of the left-behind village population. Across the 11-year study period, a major motivation for continuing life and agriculture in the settlements was family tradition. Livelihood and income-earning were already less relevant reasons to stay in 2007 and completely lost their importance in 2018. Already in 2007, 87% of the HH secured their income through pension payments, a share increasing to 98% in 2018. While this development reflects an ageing village population, it also indicates massive buildup of the government-sponsored tertiary sector [42] with its advantages for individuals and families and high burden for public finances [43, 44]. This notion is further supported by the fact that the share of HH with members involved in private sector businesses increased from 3% to 15% during the 11-year study period as a consequence of the successful development of government-supported small-scale entrepreneurship [43]. Similar trajectories have been observed in European high nature-value farmlands [6, 13].During the study period, the share of HH possessing basic information and communication assets increased, as did the share of those with a bank account. The latter mirrors the increasing role of pension payments for which an official account is needed, as well as the increasing involvement in business [42, 43]. The stagnating share of HH possessing a car reflects the fact that a typical family only uses one car. Moreover, there is possibility to travel to Sayh Qatanah and Nizwa with the “schoolbus” (four-wheel drive vehicles that transport school children to Sayh Qatanah and back every day). While in 2007, basic needs such as food and clothing constituted the major household expenditures, expenses for housing became most important in 2018. The local population’s quest for spacious houses with modern amenities such as kitchens, bathrooms, and air conditioning was already noticeable in 2007 and gained momentum during the last decade [1, 23].

### Agricultural development and product marketing

Oasis agriculture in Oman has always been characterized by small land holdings per HH, for which multi-storey systems, a three to four-fold annual crop rotation, high input of animal manure, and a judicious irrigation schedule compensated [17, 19]. These centuries-old practices resulted in high crop yields and water use efficiency, and granted food self-sufficiency of oases dwellers as long as their numbers were low and consumption patterns were modest [17, 20]. This has considerably changed during the past two to three decades [45]. Managed mosaic landscapes such as these terraced agro-forest gardens are the result of complex co-evolutionary processes within agricultural systems; they are often extremely sustainable and provide niches for *in situ* conservation of old cultivars [8, 46] as well as wild plants [5] and birds [47]. During the eleven years covered by the two survey rounds, changes in the area of farmland managed per HH were not perceivable, with renting out or leasing land, respectively, being infrequent practices and concerning only very small areas or individual trees and rose bushes. However, the share of farmers who confirmed that a substantial part of their land lay fallow increased from 34% to 54%. This was backed by the longitudinal 2007|2018 GIS data according to which fallowed and abandoned surfaces, respectively, increased by 62% and 5% across Wadi Muaydin [23].

Across the years, farmers in Wadi Muaydin focused on cash crops, namely pomegranate, rose (S, Q) and date (M) plus other temperate (S, Q) or subtropical fruits (Q, M), depending on altitude. This was confirmed by the physical crop inventory conducted in 2018 [23]. Although statistical significance was not obtained in any case, the share of HH using machinery and agrochemicals, pruning trees, and collecting and applying animal manure increased during the 11-year study period. Furthermore, the yearly quantity of applied manure doubled, with some HH even purchasing additional manure from villages on the Sayq Plateau in 2018. While a rising focus on cash crops and an intensifying use of inputs are phenomena also reported from oases in the Adrar region of Algeria [31], the continuation of traditional pruning and organic manuring practices point to farmers holding on to local knowledge: without continued manure application, soil fertility of the irragic anthrosols would rapidly decline [1].

Problems notoriously mentioned in the interviews were shortage of irrigation water and occurrence of pests and diseases in the crop and livestock sector. The respondents’ increasing recourse to pesticides indicates growing problems with plant diseases, a multi-factorial challenge often related to agricultural intensification, increasing ambient temperatures, lower water availability, and interregional mobility of people and commodities [48, 49]. The combined effects of climate change [29, 30] and increasing water demand of the resident population and tourists in Sayh Qatanah on the availability of irrigation water in the Wadi Muaydin settlements have already been cautioned against for some time [23, 50]. The sewage plant in S that provides greywater for irrigation, numerous rainwater dams, reservoirs, and bore wells on the Sayq Plateau, and the recent opening of a pipeline for desalinated water from the coast to Jabal Akhdar prove the government’s efforts to alleviate the problem [22, 23], which severely threatens the continuation of the regional oasis agriculture. Another government initiative that started in the 1990s was the promotion of drought-tolerant olive trees at higher altitudes and partial replacement of the traditional *falaj* irrigation by drip irrigation [36]. During an initial phase, tree seedlings were given out for free; afterwards, investment in planting material, terrace structures, and irrigation infrastructure largely relied on private investments (M. Al-Rawahi, pers. comm. January 2022). While in 2007, olive tree cultivation was rare on Jabal Akhdar, it started to gain momentum in 2018.

Although goats maintained their importance in terms of the share of HH keeping them, their number decreased by an average of 7 head per HH over the past three decades. During the same period, the number of sheep increased by an average of 2.6 head per HH. While in 2007, the holiday of *Eid-al-Kabir* was in mid-December (about ten months after interviews were conducted), in 2018 it was in August (about five months after interviews were conducted). This may explain the substantially higher total cattle number in the three settlement in 2018 (n = 47) as compared to 2007 (n = 19), because at least in some families, cattle are not continuously kept, but young animals are bought and fattened for this holiday.

A remarkable increase in the share of chicken-keeping HH from less than 20% to 65% occurred in the 11-year time span, accompanied by a substantial increase in flock size. This agrees with observations of Al-Qamashoui and colleagues [38] in Oman and the global trend towards extensive or semi-intensive backyard chicken keeping by urbanized households [51]. Overall, the data show that the purpose of livestock keeping shifted from supplying households with meat, milk, eggs, dung, and fiber to primarily meat and dung provision, which is a typical indicator of agricultural intensification [52]. At the same time, the high importance of manure collection and application points to farmers’ preservation of close crop-livestock integration as a cornerstone of sustainable oases farming [26, 52]. However, the increased use of unsold bakery products (energy supplements) in goat feeding and the declining practice of feeding dried sardines and date fruits (protein and energy supplements) that characterized Oman’s small ruminant husbandry for centuries [53, 54] point to changes in livestock management. This change may in parts be related to high (transport) costs for fish and date fruits that were already identified as expensive feeds in 2007 [37]. Also, raising numbers of urban dwellers and tourists on the Sayh Plateau increased the number of shops and hotels ([23]; authors’ own observations) and facilitate access to food waste such as unsold bread [55]. Another important change is the absence of alfalfa cultivation in 2018, which might be due to its high demand for irrigation water and labour [14]. A further reason is the limited suitability of alfalfa as an under-storey crop in tree plantations where fodder plants such as Rhodes grass, green oats, and green barley predominate [23], and the increasing reliance on forage imported from the Al Batinah lowlands [15].

Across the study period, small ruminant herding on mountain pastures remained an important part of animal-keeping routines. In line with the insights on arable farming, this is a clear indicator that also in livestock farming, innovations and traditional management aspects are combined to ensure the health of the overall system and the individual animal, by allowing for daily exercise outside enclosures and access to a wide variety of fodder plants on the rangelands [56]. At the same time, farmers’ main problems related to animal husbandry continued to be the time needed for feeding, herding, cleaning, and fodder cultivation and harvest. Similarly, the physical workload in cropping and animal husbandry remained a predominant problem. Although in 2007 and 2018, annual crop and fodder cultivation, irrigation tasks, and herding were mentioned among the most labourious tasks, monitoring of hired labourers advanced to an important and time-consuming task in 2018. This points to a strong increase in short- or long-term engagement of migrant labour in Oman [57] as another substantial change in farm management practices over time. The shift from sporadically hiring day-workers to a quarterly or half-yearly employment of workers (58% of HH in 2007 *versus* 89% of HH in 2018) speaks against oasis agriculture being pursued as a leisure-time activity. Since in addition to the money paid in cash the workers received in-kind benefits (meals, accommodation), it cannot be assessed if the 2018 remunerations were in agreement with the minimum wage level of monthly 225 Omani Rials [https://www.minimum-wage.org/international/oman]. Yet, it was in any case far higher than the minimum wages -for agricultural labour- in the workers’ home countries, namely Bangladesh, northern India, and Pakistan [https://www.minimum-wage.org/international/ Bangladesh;…/india; …/pakistan]. Increased recurrence to migrant labour is also reported from intensifying oasis systems in Algeria, whereby workers in the Adrar region mainly migrate within the country [31]. Replacing resident agricultural family labour by employed foreign workers may entail a loss of traditional knowledge and management skills, and can challenge system sustainability [2, 52, 58]. Across the globe, oasis inhabitants have acquired detailed agro-ecological knowledge over time, and the systems’ perpetuation relies, among others, on location-specific, inter-generational knowledge transfer [46]. In the Jabal Akhdar region, the latter practice is endangered by the out-migration of younger family members that results in the observed ageing oasis population.

Agricultural product processing was of little importance in both survey years, and the major products, vinegar and rose water, were of simple nature. The share of HH engaged in product sales slightly decreased from 2007 to 2018, with an increasing importance of farm-gate sales and a decreasing importance of sales to shops in Sayh Qatanah and at the Nizwa market. Government-promoted olive tree cultivation, fruit pressing, and oil marketing [36] has not yet gained wide acceptance. This resembles reports from the Adrar region in Algeria, where agricultural practice modernized only gradually in traditional oases settlements, whereas large-scale greenhouse vegetable production developed in neighboring government-supported new locations [31].

### The future of oasis agriculture

In both study years, farmers strongly expressed the wish to maintain their agricultural activities as part of family tradition and personal and cultural identity, an observation also reported for the inhabitants of the low mountain range region of Transylvania, Romania [2]. For the 28 HH who participated in both survey rounds, their anticipated (2007) and realized (2018) future matched surprisingly well. However, while in 2007, a majority of interviewees was optimistic about the future of their farming operation, this changed to a predominantly pessimistic view in 2018. Already in 2007, farmers were quite reluctant to embrace tourism as a major avenue for jobs and income, mainly because of potential threats to their social-ecological system [37]; this did not change until 2018. The recent establishment of high-class hotels [59] that employ low-wage personnel from South and Southeast Asia and English-speaking young males from Oman’s lowlands rather than local youth as tour guides ([60]; authors’ own experiences) adds to the ambivalent feelings of local residents.

In view of the above, it can be maintained that until today, central elements of sustainable oasis agriculture, namely use of crop rotations, herding livestock on mountain ranges, and close crop-livestock integration with its central component of organic manure application to terraced farmland, are still in place. Even though these practices are increasingly executed by hired foreign workforce, the high amounts of manure applied, the perpetuated quality herding of animals, the relatively low absolute extent of abandoned and fallowed surfaces, the increasing focus on cash crop cultivation in parallel to a still high agro-biodiversity point to the farmers’ dedication to maintain their farming system while adapting it to current socio-economic conditions and possibilities. Hybridization of old and modern management approaches such as use of “non-halal” treated wastewater for crop irrigation, and the renovation of terrace walls and ancient irrigation channels with cement rather than traditional *sarooj* mortar [23] indicate, however, limits to the system’s inherent elasticity against alienation of cultural identity [61]. While well-managed hybridization can ensure the further existence of oasis settlements as demonstrated by examples from Algeria [31, 62], the re-construction of terrace walls and irrigation channels with cement in our study region has already led to the loss of sustainability indicators such as the endemic wild species *Campanula akhdarensis* A.G. Mill. & Whitc., of which the worldwide largest population had been found in S, but has completely disappeared during the last years ([63]; A. Patzelt, pers. comm. May 2021). The same has happened for ancient germplasm of wheat which once played a major role in mountain oases of Oman [8, 64, 65]. This demonstrates that the ecological preservation of the high nature-value oases farmlands is at threat [2, 11, 23], even though the pathway of partial modernisation of mountain oasis agriculture allowed more than half of the local HH to continue their farming activities until present. However, even a transforming oasis agriculture will have little future in the Hajar Mountains without new reliable and lucrative marketing of primary and processed agricultural products by local farmers. To this end, the buildup of profitable value chains for local oasis products is indispensable [16, 33]. Thoughtful consideration and implementation of concepts such as geographical labeling for animal- and plant-based food originating from its mountain oases in combination with community-based ecotourism [66] could be an important step towards preserving Oman’s “Hanging Gardens” and the traditional cultural identity of their inhabitants.

## Conclusions

Like other agricultural systems in mountainous or island regions, oases settlements in the northern Hajar Mountains of Oman are undergoing major land-use transformations typical for many high-nature value farmlands worldwide. As elsewhere, their cultivation increasingly relies on culturally foreign, largely unskilled, migrant labour. While some of the traditional agricultural practices that secured the oases’ long-term persistence over centuries are still in use, political support is needed to foster their continued existence as social-ecological model systems whose sustainability has stood the test of time. To support local farmers in their endeavors to continue practicing mixed irrigated agriculture on the steep slopes of Jabal Akhdar as part of their identity, the establishment and use of profitable value chains for primary and processed agricultural products in combination with sustainable forms of ecotourism seem an option worthwhile to pursue.
